# A Case of Pancreatic Duct Leak Presenting as Lower Extremity Pain and Edema

**DOI:** 10.7759/cureus.18839

**Published:** 2021-10-17

**Authors:** Mukunthan Murthi, Abdulrahman Abusalim, Zohaib Haque, Christine Acob

**Affiliations:** 1 Internal Medicine, John H. Stroger, Jr. Hospital of Cook County, Chicago, USA

**Keywords:** persistent leg pain, unilateral leg swelling, acute-on-chronic pancreatitis, duct leak, pancreatic duct

## Abstract

Pancreatic pseudocyst from pancreatic duct leak is one of the common complications of both acute and chronic pancreatitis. The presentation of such leaks can range from patients being completely asymptomatic to septic shock. Extra-abdominal collections of fluid due to pancreatic duct leaks are very rare. We describe a diagnostically challenging patient with acute chronic pancreatitis presenting with lower extremity swelling and pain, who was found to have a pancreatic fluid leak into the right iliopsoas, right gluteal, and thigh muscle compartment. Despite endoscopic stenting of the pancreatic duct and multiple percutaneous drain placement and antibiotic treatment for abscess formation, the patient's condition deteriorated clinically. Surgical options could not be pursued due to poor functional status.

## Introduction

Pancreatic duct leak may arise from acute and chronic pancreatitis, or as a complication of surgery or trauma. The progression of pancreatic duct leak can result in the development of various clinical entities including pseudocyst formation, pleural effusion, ascites, and pancreatic fistulas. Among them, pancreatic pseudocyst is the most common, occurring in 30-40% of patients with chronic pancreatitis [1]. In patients with chronic pancreatitis, the leakage typically occurs due to stricture development or intraductal stones [2]. Pancreatic duct leak resulting in fluid collections outside of the abdomen is rare. We describe a case of pancreatic duct leak resulting in an iliopsoas and thigh fluid collection presenting with pain and edema of the right lower extremity.

## Case presentation

**Figure 1:**
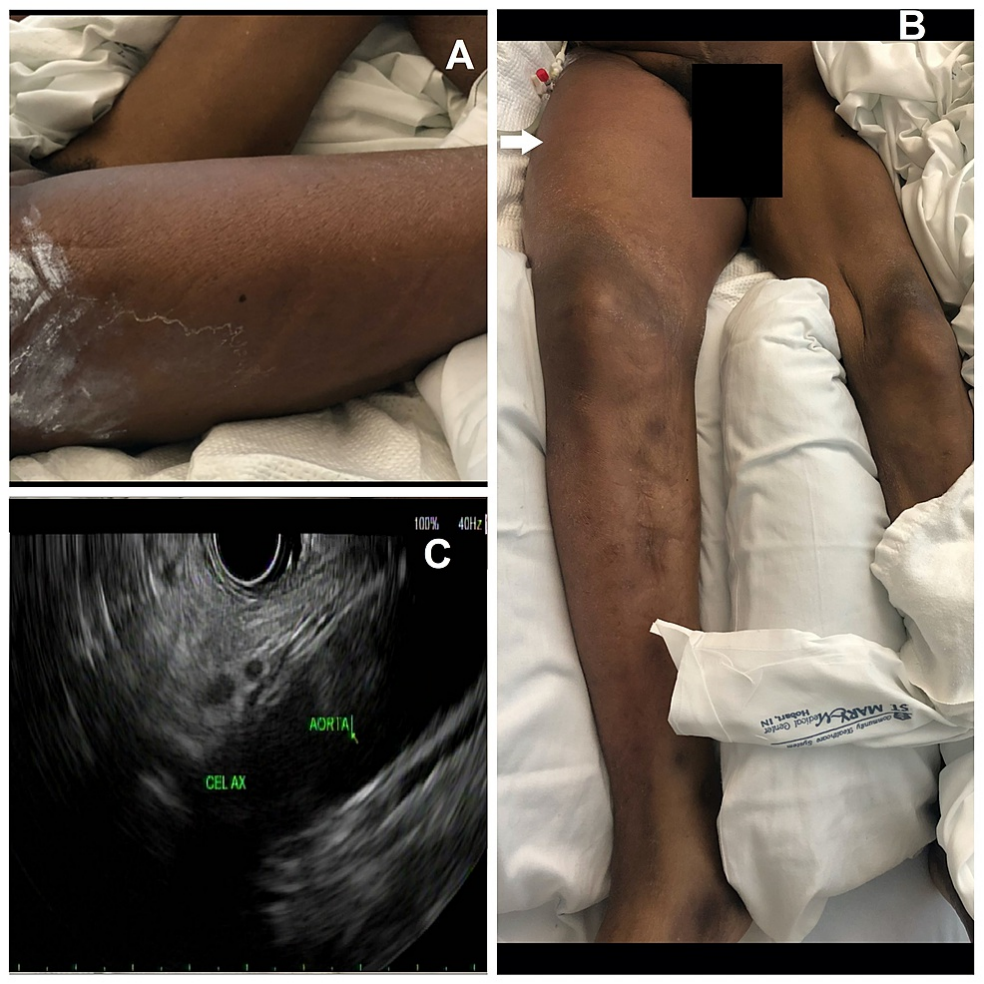
A and B: Images showing right thigh and gluteal region swelling with associated erythema. C: EUS showing diffusely hypoechoic pancreas with lobularity and multiple linear and focal areas of echogenicity suggestive of the atrophic pancreas with calcifications. EUS, endoscopic ultrasound.

A 58-year-old man presented with a one-week history of progressively worsening bilateral lower extremity swelling and pain accompanied by non-bloody, non-bilious vomiting for two days. The patient had no past medical or surgical history, but his social history was significant for chronic alcohol abuse with consumption of 1 pint of alcohol/day for many years. On physical exam, the patient was tachycardic to 114/min and had mild abdominal distention with diffuse tenderness. The patient had left lower extremity edema extending from his ankle up to his knee and right lower extremity swelling extending from his gluteal region to below his right knee, with overlying erythema, warmth, and tenderness (Figure 1).

**Figure 2:**
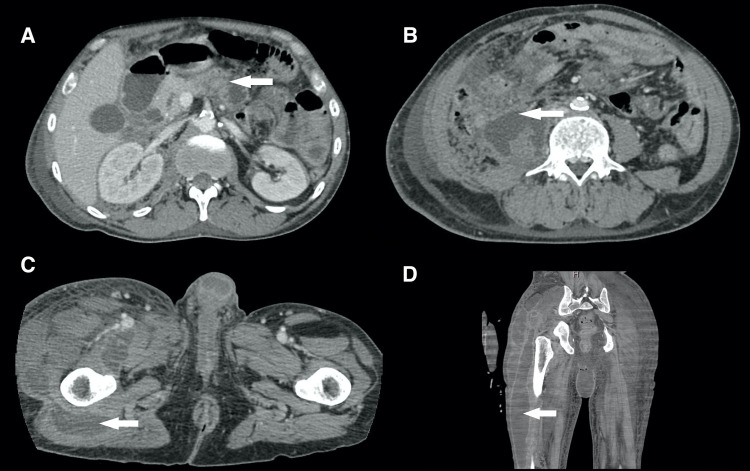
A: Sequela of chronic pancreatitis with pancreatic proximal calcifications and atrophy with the dilated pancreatic duct. B: Right psoas abscess measuring 5.6 x 3.8 cm. C: Gluteal musculature abscess measuring 5.7 x 2.2 cm. D: Fluid extending through the lateral thigh compartment.

Lab results were significant for an elevated WBC count of 14.8 cells/dL (reference range 4.4-10.6 cells/dL) and lipase level of more than 5,000 U/L (reference 5-55 U/L). Due to concern for acute pancreatitis, CT of the abdomen and pelvis was obtained, which showed pancreatic calcifications and associated intrahepatic and extrahepatic biliary ductal dilation, as well as main duct dilatation with stones seen in the gallbladder. In addition to these findings, a large 5.6 x 3.8 cm multiloculated abscess collection involving the right iliopsoas and multiple right gluteal musculature abscesses, the largest of which was 5.7 x 2.2 cm in the gluteus maximus, was noted. Both of the aforementioned collections were interconnected (Figure 2). Deep vein thrombosis in the left common femoral vein was also seen, which was possibly secondary to the inflammatory state of pancreatitis and the patient was started on heparin for anticoagulation. Subsequently, the patient underwent ultrasound-guided aspiration of fluid from the pocket near the right trochanter. Subsequent gram stain, cell count, and culture were all unremarkable.

On day two of admission, CT-guided right lateral flank, percutaneous 10 French locking pigtail drainage of the multiloculated right iliopsoas abscess was performed. The aspirate consisted of watery bloody fluid, which did not have any microorganisms. Over the next few days, the drain continued to draw great than 1 liter of clear straw-colored fluid per day. The drain fluid was sent for analyses and amylase and lipase were found to be elevated >12,000 U/L and >6,000 U/L, respectively. Hence, a diagnosis of pancreatic fluid leak into the psoas muscle and right thigh muscles was made. An endoscopic retrograde cholangiopancreatography (ERCP) with EUS (Figure 1) showed the pancreas was diffusely hypoechoic with lobularity and had multiple linear and focal areas of echogenicity suggestive of the atrophic pancreas with calcifications and a dilated pancreatic duct at 4.1 mm. An occlusion cholangiogram demonstrated dilation of the common bile duct to 11 mm, with distal common bile duct (CBD) filling defects. It also demonstrated saccular dilation of the left common hepatic ducts, raising suspicion for Caroli syndrome, a congenital disorder characterized by multifocal, segmental dilatation of large intrahepatic bile ducts. On pancreatogram, contrast filling of the distal pancreatic duct at the level of the tail of the pancreas was not well visualized and subsequently, a 4 Fr single pigtail Zimmon plastic stent was placed. However, no obvious site of the leak was identified. Postcontrast imaging on magnetic resonance cholangiopancreatography (MRCP) was also non-diagnostic. Despite continued drainage and octreotide treatment, repeat imaging on day 15 of hospitalization showed increased fluid collections in the musculature of the right hemipelvis and proximal right thigh. Repeat CT-guided catheter placement was done in the iliopsoas region, which drained the blood-tinged serous fluid and a new drain was placed in the right lateral hip tensor lateral fascia-gluteus medius muscle, which drained frank pus. Cultures showed growth of *Pseudomonas aeruginosa*, *Klebsiella pneumoniae*, and *Hafnia alvei*, and the patient was treated with antibiotics per susceptibilities. Despite additional drain placement in the thigh and appropriate antibiotic therapy, the patient’s condition continued to worsen. Repeat imaging showed worsening of fluid collection in the gluteus and right thigh muscular compartment. Surgical intervention was deferred due to poor functional and nutritional status and a high risk of complications. Eventually, on day 25 of admission, the patient was transferred to a skilled nursing facility with hospice care.

## Discussion

Patients with chronic pancreatitis may be asymptomatic or may develop recurrent episodes of acute pancreatitis in the early course of the disease [3]. Pseudocyst development is a common clinical entity in patients with both acute and chronic pancreatitis. Alcohol-induced pancreatitis has a higher tendency of such pseudocyst development compared to other causes [4]. The presentation can vary widely, ranging from completely asymptomatic to sepsis resulting from infected fluid collections. Diagnosis is often challenging given only two-thirds of the patients have a demonstrable connection between the cyst and the pancreas [1].

Presentation of patients with extra-abdominal signs and symptoms is extremely rare. Few cases of pancreatic pseudocyst presenting as groin mass and hernia-like lesions have been previously reported [5-7]. Mundra et al. described a pancreatic pseudocyst presenting with dyspareunia and inguinal mass but normal blood levels of pancreatic enzymes [8]. Very few case reports have described pancreatitis resulting in fluid collections in the lower limb. Lye et al. described a patient with pancreatic pseudocyst extension into the thigh up to the patella, which was successfully treated with multiple drain placements [9]. Shimizu et al. elaborated on a patient with rupture of intraductal papillary mucinous neoplasm (IPMN) of the pancreas resulting in extension of fistula into the thigh. Similar to this report, their patient did not report any abdominal pain and only had thigh swelling and elevated pancreatic enzymes [10]. The wide plethora of presentations makes the diagnosis particularly challenging and can result in significant delays of appropriate treatment.

Most asymptomatic patients with duct leaks can be treated conservatively with gut rest, octreotide, and total parenteral nutrition (TPN) resulting in complete resolution. Symptomatic patients, especially those with the possibility of infected fluid collections, require drainage through endoscopic, percutaneous, or surgical procedures [1]. As the exact location of the duct leak could not be identified, endoscopic stent placement in our patient did not resolve the leak and our patient continued to drain fluid despite multiple percutaneous drain placements. Our patient would have ideally undergone surgical management including abdomen exploration and incision and drainage for the abscess but was unfortunately not medically fit for such interventions.

## Conclusions

Our case elaborated how pancreatic duct leak can have a widely variable presentation. The constellation of unusual symptoms made our patient a diagnostic challenge. Our case report emphasizes the high degree of suspicion that is required to diagnose and promptly treat uncommon presentations of pancreatic duct leaks. Although asymptomatic patients can be treated with conservative management, endoscopic, percutaneous, and surgical therapies may be required for symptomatic patients.
